# The electrostatic potential of dynamic charge densities

**DOI:** 10.1107/S1600576717013802

**Published:** 2017-10-20

**Authors:** Christian B. Hübschle, Sander van Smaalen

**Affiliations:** a Laboratory of Crystallography, University of Bayreuth, 95440 Bayreuth, Germany

**Keywords:** electrostatic potential, charge density, electron density, X-ray diffraction, multipole model

## Abstract

A procedure to derive the electrostatic potential (ESP) for dynamic charge densities obtained from structure models or maximum-entropy densities is introduced. The ESP essentially is obtained by inverse Fourier transform of the dynamic structure factors of the total charge density corresponding to the independent atom model, the multipole model or maximum-entropy densities, employing dedicated software that will be part of the *BayMEM* software package. Our approach is also discussed with respect to the Ewald summation method. It is argued that a meaningful ESP can only be obtained if identical thermal smearing is applied to the nuclear (positive) and electronic (negative) parts of the dynamic charge densities. The method is applied to structure models of dl-serine at three different temperatures of 20, 100 and 298 K. The ESP at locations near the atomic nuclei exhibits a drastic reduction with increasing temperature, the largest difference between the ESP from the static charge density and the ESP of the dynamic charge density being at *T* = 20 K. These features demonstrate that zero-point vibrations are sufficient for changing the spiky nature of the ESP at the nuclei into finite values. On 0.5 e Å^−3^ isosurfaces of the electron densities (taken as the molecular surface relevant to intermolecular interactions), the dynamic ESP is surprisingly similar at all temperatures, while the static ESP of a single molecule has a slightly larger range and is shifted towards positive potential values.

## Abstract 2

The electrostatic potential (ESP) is computed for dynamic charge densities corresponding to multipole models and maximum-entropy densities. Convergence of the reciprocal-space summation is guaranteed by the Gaussian form of the Debye–Waller factor. Applications to serine demonstrate only a weak temperature dependence of the ESP on molecular surfaces relevant to intermolecular interactions.

## 1. Introduction   

The electrostatic potential (ESP) is important for understanding the chemical reactivity and the atomic structure of molecules and solids. A variety of properties can be derived from the ESP, for example atomic and anionic radii, electronegativities, and energies (Politzer & Murray, 2002 ▸).

The ESP is most easily computed for a single molecule or finite cluster of atoms, for which a well defined electron density is available. This is the case for the static electron densities obtained by molecular quantum chemical methods (Kumar *et al.*, 2015 ▸). Considerable effort has been devoted to the development of methods for calculating the ESP from the static electron density of an isolated molecule, which is described by the multipole (MP) model (Stewart, 1976 ▸; Hansen & Coppens, 1978 ▸) as it can be extracted from a crystal structure (Su & Coppens, 1992 ▸; Ghermani *et al.*, 1993 ▸; Stewart & Craven, 1993 ▸; Volkov *et al.*, 2006 ▸). One method of analysis comprises the consideration of the ESP on a surface enveloping the molecule. In applications to small and large mol­ecules up to proteins, the ESP has thus been used to identify electrophilic and nucleophilic sides, to characterize hydrogen bonds, and to analyse intermolecular interactions (Du *et al.*, 2016 ▸; Kalaiarasi *et al.*, 2016 ▸; Kirby *et al.*, 2014 ▸; Malinska & Dauter, 2016 ▸; Niranjana Devi *et al.*, 2017 ▸; Sirohiwal *et al.*, 2017 ▸; Zarychta *et al.*, 2015 ▸; Zhurova *et al.*, 2016 ▸).

The ESP of an infinite crystal is not uniquely defined. For example, the correct ESP requires that all finite approximations to the infinite sum pertain to electrically neutral crystals, for instance the summation needs to be performed over complete unit cells. One solution to this problem is the Ewald summation method (Ewald, 1921 ▸), which combines converging sums in direct and reciprocal spaces. Earlier methods of evaluating the ESP in crystals involve its computation directly from the X-ray diffraction data (Bertaut, 1952 ▸, 1978 ▸; Stewart, 1979 ▸). These methods tend to suffer from series termination effects of the Fourier sums. In other methods the thermal averaged deformation density is used in combination with the ESP of the static independent atom model (IAM), resulting in an ESP approximately valid for static densities of crystals (Spackman & Stewart, 1981 ▸; Spackman & Weber, 1988 ▸; Brown & Spackman, 1994 ▸; Spackman, 2007 ▸; Franchini *et al*., 2014 ▸).

Tanaka *et al.* (2006 ▸) have computed the ESP from an electron density obtained by the maximum entropy method (MEM) applied to X-ray diffraction data. Owing to the dynamic character of this density, the reciprocal-space sum of structure factors converges. The nuclear contribution is computed by Ewald summation involving the positions of the nuclei (Tanaka *et al.*, 2009 ▸; Fujiwara *et al.*, 2012 ▸). This method results in an ESP that combines a dynamic electron density with a static nuclear density.

Here we propose a method of computation of the ESP for dynamic charge densities inside a crystal. The method basically involves the inverse Fourier transform of the structure factors of the total charge density. However, instead of experimental structure factors, the structure factors of a model are employed, which then are required up to resolutions of at least 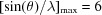
 Å^−1^ in order to reach convergence. Presently, we can compute the dynamic structure factors of the MP model and IAM as well as of MEM densities.

## 2. The electrostatic potential in direct and reciprocal spaces   

The ESP 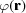 at position 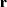 due to a charge 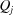 at position 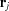 is defined as (Coppens, 1997 ▸)
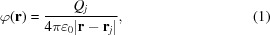
where ∊_0_ is the permittivity of free space. A charge density 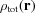 is defined in units of elementary charge per volume as the difference between proton and electron densities,
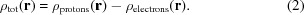
For a collection of atoms or pseudoatoms with atomic numbers 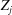 and static electron densities 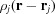 centred at positions 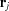, the total charge density can be expressed by a sum over all atoms 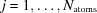 in the crystal,
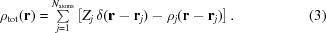
This charge distribution leads to the electrostatic potential for static charge distributions,
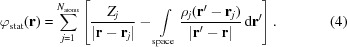
A periodic structure has 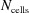 unit cells, each filled with 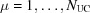 atoms, such that 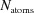 = 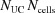. In analogy to the structure factor of electrons, the structure factor 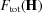 of the total charge density is defined as
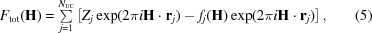
where 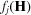 is the aspherical atomic scattering factor of atom *j* (Coppens, 1997 ▸). The total charge density of a periodic structure can then be expressed as the inverse Fourier transform of its structure factors, where the latter are defined on the nodes 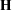 of the reciprocal lattice and 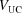 is the volume of the unit cell:
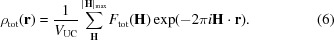
The summation extends up to an upper limit 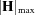, which is chosen to be sufficiently large for convergence to have been reached. The ESP can also be expressed as an inverse Fourier transform involving the structure factors of the total charge density,
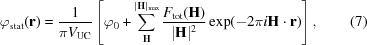
where the term 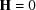 is excluded from the summation.

In the limit of arbitrarily large crystals (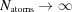 and 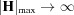), both expressions for 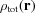 converge to the same values [equations (3) and (6)]. The same is true for the direct- and reciprocal-space expressions for the ESP [equations (4) and (7)]. However, convergence is too slow.

The convergence problem has been solved by Ewald (1921 ▸) in what has become known as the Ewald summation method. In this method the ESP of the total charge density is obtained as the sum of direct-space and reciprocal-space contributions,
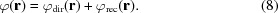
Each of the two contributions is described by a rapidly converging series,
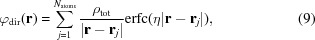


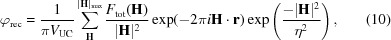
where 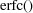 is the error function and the single adjustable parameter η should be chosen such that both sums rapidly converge.

It is noted that the product 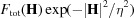 actually is the structure factor of a dynamic charge density, whereby each atom has been assigned the same isotropic displacement parameter 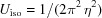. This observation leads to the conjecture that equation (7) for 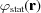 will converge sufficiently rapidly if 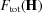 is replaced by the structure factor of the dynamic charge density [compare with equation (5)],
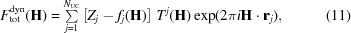
where 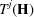 is the Debye–Waller factor of atom *j*. For example, for anisotropic, harmonic atomic displacement parameters it is
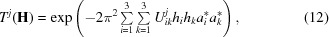
where 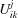 are the anisotropic displacement parameters of atom *j*. Substitution of 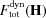 into equation (7) leads to an expression for the ESP 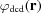 of dynamic charge densities,
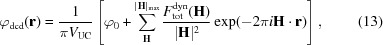
where the term 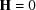 is excluded from the summation. The macroscopic contribution 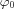 to the ESP is (Becker & Coppens, 1990 ▸)
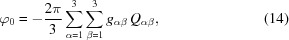


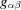
 being the metric tensor and 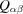 the quadrupolar tensor obtained by the summation rules given by Becker & Coppens (1990 ▸).

The same idea has been used to compute dynamic electron densities by Fourier inversion of dynamic structure factors (Mondal *et al.*, 2012 ▸). Convergence has been demonstrated for sums that include all structure factors up to 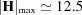
 Å^−1^, even in cases where atomic displacement parameters basically represent zero-point vibrations (Mondal *et al.*, 2012 ▸). Owing to the additional factor of 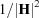, a more rapid convergence is expected for the ESP [equation (11)]. Present calculations confirm this convergence behaviour.

If the dynamic electron density is available through its values over the unit cell, as is the case for electron densities 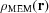 obtained by the MEM, the dynamic structure factor is [equation (11)]
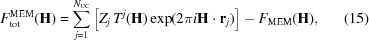
where 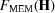 is the Fourier transform of 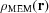. The corresponding ESP follows by substitution of 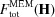 into equation (13)).

Within the present approach the Debye–Waller factor is responsible for convergence of the reciprocal-space summation [equation (13)]. On the other hand the Debye–Waller factor represents thermal motion of the atoms. In order to arrive at the ESP of a dynamic charge density it is thus important to employ for each contributing atom the same position and the same atomic displacement parameter for its nucleus and its electron density [equation (11)]. Non-matching values for atomic displacement parameters of nuclei and electron densities lead to an apparent ESP without a clear physical meaning. The latter function may then contain artefacts like Fourier ripples. The choice of matching atomic displacement parameters is implicit in equations (11) and (13), and it appears the logical choice for dynamic ESPs to be based on structure models, like the multipole model and the IAM. This requirement poses a challenge for dynamic electron densities that do not originate from a model, but are, for example, obtained by the MEM. Here the model best matching to the MEM electron density should be used in equation (15). These aspects are discussed in §3.2.2.

## 3. Computational details   

### 3.1. Details of the algorithm   

Following earlier work on the dynamic electron density and the MEM, the electron density is described by its values on a grid over the unit cell (van Smaalen *et al.*, 2003 ▸; Mondal *et al.*, 2012 ▸). Structure factors at scattering vectors 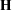 follow by discrete Fourier transform from the electron density, employing the fast Fourier transform (FFT) algorithm. The resolution 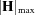 in reciprocal space is inversely proportional to the mesh of the grid in direct space. Convergence of the Fourier transform and its inverse is obtained for a mesh better than ∼0.04 Å, corresponding to 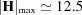
 Å^−1^ (Mondal *et al.*, 2012 ▸).

In a first step a dynamic model electron density is produced by the software *PRIOR* for an MP model or an IAM, or a MEM density is obtained by the *BayMEM* software (van Smaalen *et al.*, 2003 ▸). Of course, gridded dynamic electron densities from other sources, including theoretical densities, can be used as well.

A new software, *dESP*, has been written, which employs this dynamic electron density together with atomic coordinates and displacement parameters for generating the ESP of the dynamic charge density according to equation (11). The program *dESP* will be part of the *BayMEM* suite.

The computation involves the following steps:

(1) Load the gridded dynamic electron density.

(2) Apply the FFT to produce the structure factors.

(3) For each scattering vector 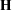 of the grid, calculate the thermally smeared nuclear structure factor from atomic coordinates and displacement parameters.

(4) Replace the structure factor by the difference between the nuclear structure factor and the structure factor, divided by 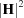 [compare equations (11) and (13)].

(5) Calculate the average potential 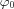 by the summation rules given by Becker & Coppens (1990 ▸), and use it as the term 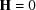.

(6) Apply the inverse FFT to produce the ESP according to equation (13).

(7) Write the ESP to a file.

### 3.2. The dynamic ESP of dl-serine at temperatures of 20, 100 and 298 K   

#### 3.2.1. Dynamic properties of multipole models   

Dittrich *et al.* (2005 ▸) have published high-resolution X-ray diffraction data of dl-serine as measured at temperatures of 20, 100 and 298 K. They reported MP models based on invariom refinements (Dittrich *et al.*, 2013 ▸) against all three datasets. Their results demonstrated a consistent description of the aspherical atomic electron densities, independent of temperature. Mondal *et al.* (2012 ▸) have reported an MP refinement against the 20 K data. Subsequently invariom-like refinements were performed against the 100 and 298 K data, where the MP parameters were kept fixed at their 20 K values and only positional parameters and atomic displacement parameters were refined. Mondal *et al.* (2012 ▸) employed these three structure models for a study of the effect of temperature on the dynamic electron density. It was found that within regions of bond-critical points (BCPs) static as well as dynamic densities possess surprisingly similar topological properties.

Here we have reproduced the refinement strategy of Mondal *et al.* (2012 ▸), arriving at multipole models MP(20), MP(100) and MP(298) for dl-serine at 20, 100 and 298 K, respectively. These MP models involve isotropic displacement parameters for H atoms. H-atom distances C—H and N—H were fixed to values from the invariom database (Dittrich *et al.*, 2013 ▸). Then for each temperature, anisotropic displacement parameters were computed for H atoms, employing the *SHADE3* server (Madsen, 2006 ▸). These values were introduced into the structure models and kept fixed during subsequent refinements. This procedure resulted in three more structure models, denoted as AH(20), AH(100) and AH(298), respectively.

Dynamic electron densities were computed for all six structure models by the software *PRIOR* (Mondal *et al.*, 2012 ▸) of the *BayMEM* suite (van Smaalen *et al.*, 2003 ▸). The ESP was obtained from these dynamic electron densities together with the corresponding structure models, employing the newly written software *dESP* (§3.1).

#### 3.2.2. Maximum entropy electron density   

The software *BayMEM* was employed for the computation of dynamic electron densities according to the MEM. MEM electron densities were generated for each of the three temperatures, employing the corresponding dynamic model electron densities, MP(20), AH(20), AH(100) and AH(298), as prior densities. A value of 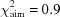 was used to define convergence of the MEM calculations. This value was the lowest value for which the MEM densities did not exhibit spurious maxima. The resulting dynamic MEM electron densities are denoted as MEMP(20), MEMAH(20), MEMAH(100) and MEMAH(298), respectively.

The ESP was computed by *dESP* for each of the four MEM electron densities, employing atomic coordinates and dis­place­ment parameters from the corresponding prior for the nuclear part. In the case of MP priors these values can be expected to be close to the unknown values hidden in the MEM density. In the case of an IAM prior significant deviations might be present, which then will lead to artefacts in the ESP.

#### 3.2.3. Quantitative measures of the ESP   

The electrostatic potential is particularly interesting in the region between atoms. For this purpose the variation of the ESP is considered on an isosurface of the electron density, which envelopes entire molecules. We have chosen the isosurface at 0.5 e Å^−3^. This value is higher than the highest value of the electron density between molecules, which is the electron density in intermolecular hydrogen bonds, with a maximum value of 0.32 e Å^−3^ in serine. And it is substantially smaller than the electron densities in covalent bonds. For visual inspection, this isosurface is provided in a pseudo-three-dimensional representation, with values of the ESP indicated by colours. Quantitative measures of the ESP have been obtained as integral properties over this surface according to Politzer *et al.* (2001 ▸) (see Table 1 ▸). Mathematical definitions are given in Appendix *A* [App appa]. Generation of the graphical representations and computations of the integral properties of ESPs have been performed by the program *MoleCoolQt* (Hübschle & Dittrich, 2011 ▸).

## 4. Results and discussion   

### 4.1. Temperature dependence of the dynamic ESP   

**Figure 1:**
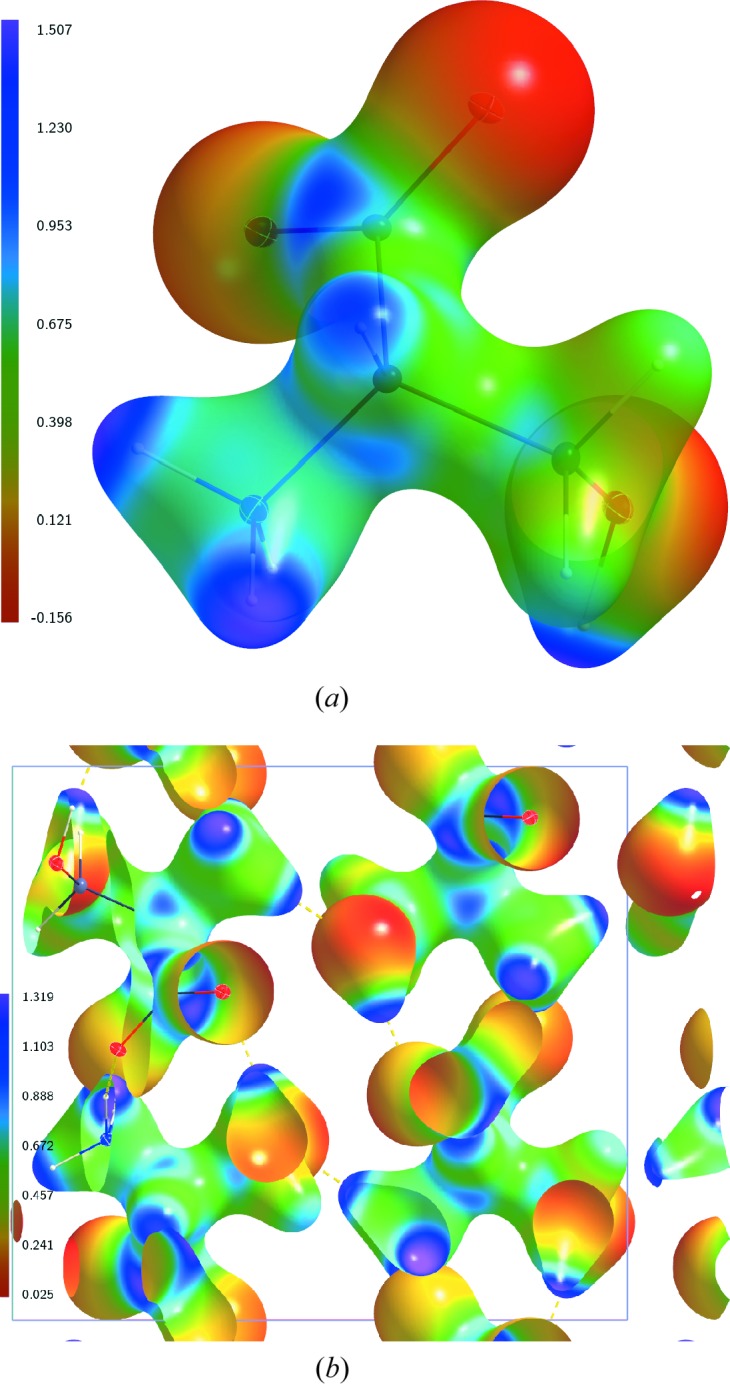
Electrostatic potential (e Å^−1^) of the static charge density of dl-serine, derived by *XDPROP* (Volkov *et al.*, 2006 ▸) from the multipole model AH(20), and mapped on the static electron density isosurface at 0.5 e Å^−3^. (*a*) ESP for a single molecule. (*b*) ESP for a cluster of 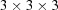 unit cells. The central unit cell is shown. The single molecule in (*a*) corresponds to the molecule in the lower-right corner of the unit cell in (*b*).

The ESP of the static charge density at 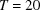
 K has been analysed for the multipole model AH(20), which includes anisotropic displacement parameters for the H atoms. On the molecular surface defined by 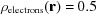
 e Å^−3^, the static ESP of a single molecule exhibits its most negative values near the three O atoms, and its most positive values near H atoms attached to nitrogen and oxygen (Fig. 1 ▸ *a*). These properties of the ESP indicate the preferred interaction sides of the serine molecule. As is actually realized in the crystal, all three O atoms act as acceptors of intermolecular hydrogen bonds, and these three H atoms are part of a hydrogen bond too (Mondal *et al.*, 2012 ▸). These features of the molecular ESP are in agreement with similar features of ESPs of other molecules (Kalaiarasi *et al.*, 2016 ▸; Niranjana Devi *et al.*, 2017 ▸; Zhurova *et al.*, 2016 ▸). The ESP of the static electron density of AH(20) has also been computed for a cluster of 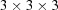 unit cells. Its values on the 0.5 e Å^−3^ isosurface in the central unit cell qualitatively exhibit the same features as the molecular ESP (Fig. 1 ▸).

**Figure 2:**
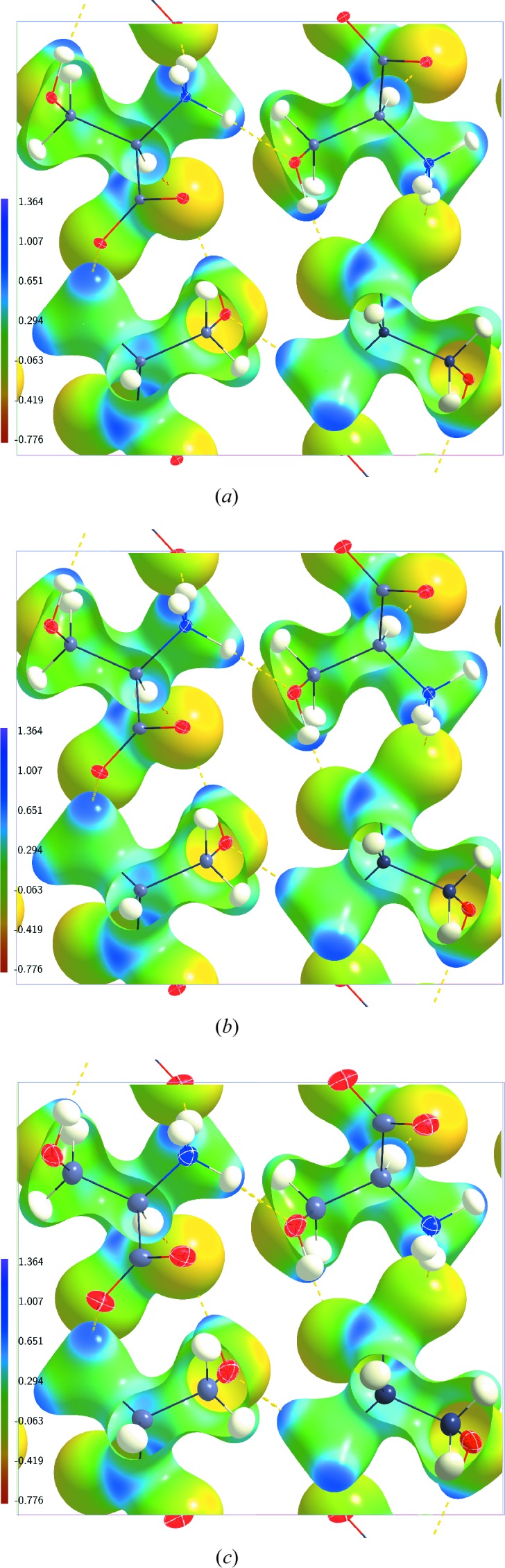
Electrostatic potential (e Å^−1^) of dynamic charge densities of dl-serine mapped on dynamic electron density isosurfaces at 0.5 e Å^−3^. (*a*) Model AH(20) at 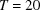
 K; (*b*) model AH(100) at 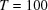
 K; (*c*) model AH(298) at 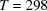
 K. The structure model with ellipsoid representation of the atomic displacement parameters of the atoms is superimposed in each case.

The ESP of the dynamic charge density inside the crystal at 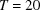
 K is given in Fig. 2 ▸(*a*) for the multipole model AH(20) on the 0.5 e Å^−3^ isosurface of the dynamic electron density. The general features of the dynamic ESP are similar to the features described above for the static cluster ESP on this isosurface. In particular, the range of values 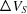 is nearly identical inside the static cluster ESP and the dynamic crystal ESP (Table 2 ▸). The absolute values 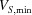 and 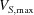 are different for these static and dynamic ESPs. This difference can be explained by the fact that the static ESP has been computed for a finite cluster, while the dynamic ESP pertains to the ESP inside a crystal. The static ESP of a single molecule has a wider range of values than the dynamic crystal ESP on their 0.5 e Å^−3^ isosurfaces. Again, this is explained by the molecular *versus* crystal character of the ESPs. It is noted that the static ESP is represented in Fig. 1 ▸ by a red-to-purple colour code encompassing its full range. A unified red-to-purple colour coding for a range of −0.776 to 1.364 e Å^−1^ has been employed for all dynamic ESPs. The value −0.776 is the lowest value and 1.364 is the highest value of the ESP on the 0.5 e Å^−3^ electron density isosurfaces of all of these ESP maps (Figs. 2 ▸–6 ▸ ▸ ▸ ▸). This unified range enables a direct visual comparison of the ESPs obtained from different dynamic electron densities.

Major differences between static and dynamic ESPs appear close to the nuclei. Static charge densities have singularities at the positions of the nuclei (Mondal *et al.*, 2012 ▸). Accordingly, the static ESPs are very large near the nuclei (Table 3 ▸). Any thermal motion – zero-point vibrations are sufficient – leads to smearing of the density and a dramatic reduction of the ESP in the neighbourhood of the atoms. Concomitantly, the electron density is increased within the low-density region between the molecules (Mondal *et al.*, 2012 ▸), leading to more negative values of the dynamic ESP than of the static ESP in its region of lowest values (Table 3 ▸).

The effect of thermal motion on the charge densities and ESPs is strengthened with increasing temperature. The maximum value of the ESP is strongly reduced on going from 20 to 100 K and again on going from 100 to 298 K [compare AH(100) and AH(298) in Table 3 ▸].

On the other hand, the dynamic ESP is nearly independent of temperature on the 0.5 e Å^−3^ isosurfaces of the electron densities (Fig. 2 ▸). This visual impression is confirmed by the integral quantities computed according to Politzer *et al.* (2001 ▸). All quantities possess nearly equal values at the three temperatures of 20, 100 and 298 K (Table 2 ▸). Only a small reduction can be observed of the variances of the dynamic ESP of AH(298).

### 4.2. Dynamic ESP for different model densities   

**Figure 3:**
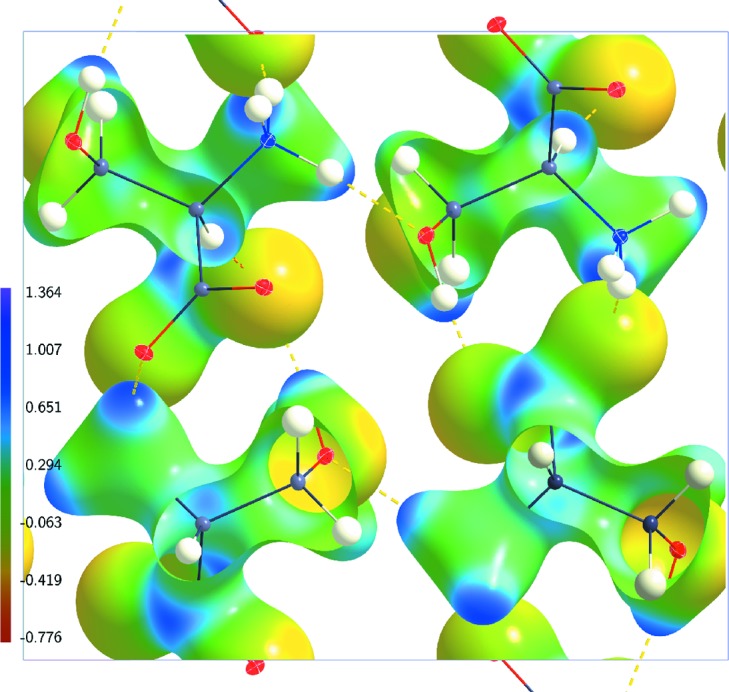
Electrostatic potential (e Å^−1^) of dynamic charge densities of dl-serine mapped on dynamic electron density isosurfaces at 0.5 e Å^−3^. Model MP(20) at 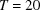
 K. The structure model with ellipsoid representation of the atomic displacement parameters of the atoms is superimposed.

We have chosen as reference the multipole model with an anisotropic description of the H atoms. An alternative choice for the multipole model employs isotropic displacement parameters for the H atoms, which then can be varied in the structure refinement. Models MP(20), MP(100) and MP(298) lead to ESPs which are close to the ESPs of the corresponding anisotropic models (Fig. 3 ▸ and Tables 2 ▸ and 3 ▸). One can thus conclude that multipole models with either isotropic or anisotropic displacement parameters for H atoms lead to similar descriptions of the ESP.

Concentrating on the dynamic ESPs at 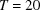
 K the IAM and invariom model have been considered. While the dynamic ESP of the invariom model is comparable to the dynamic ESP of the refined multipole model AH(20), the ESP of the IAM exhibits major differences. In particular, its range 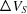 on the 0.5 e Å^−3^ isosurface is about half the range of the ESP of AH(20) (Table 2 ▸). This result is in agreement with findings for static densities of other molecules, which indicate that the multipole model is essential for extracting the correct ESP (Malinska & Dauter, 2016 ▸).

### 4.3. Dynamic ESP for MEM electron densities   

**Figure 5:**
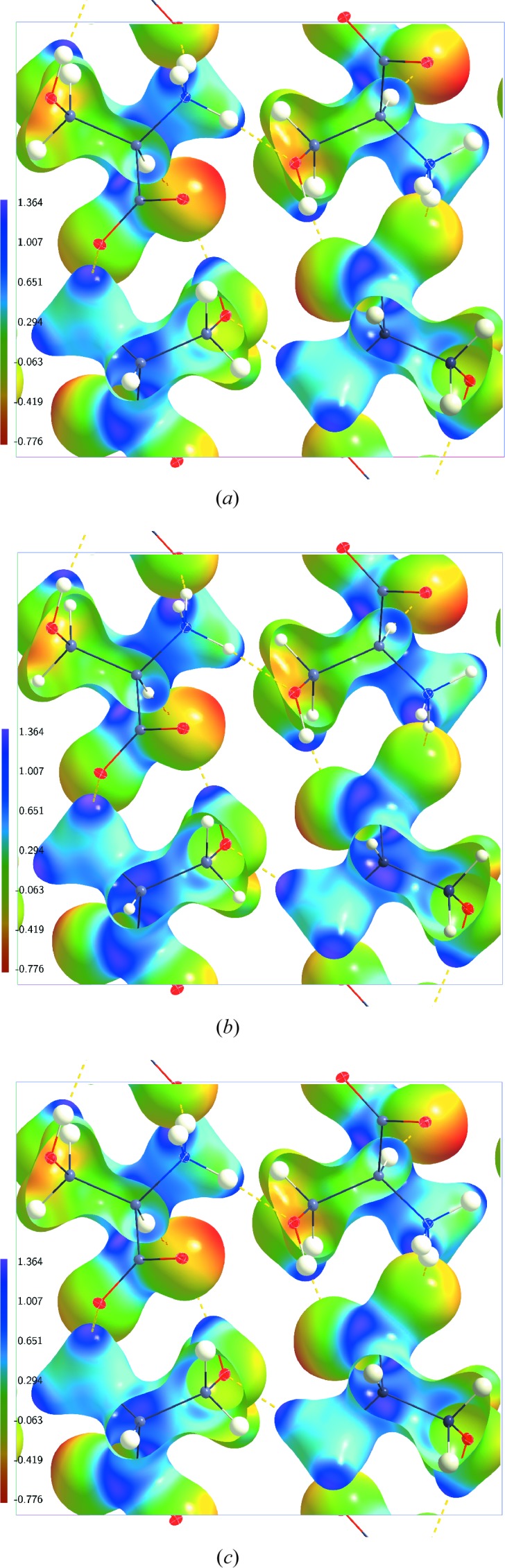
Electrostatic potential (e Å^−1^) of dynamic charge densities of dl-serine mapped on dynamic electron density isosurfaces at 0.5 e Å^−3^. (*a*) Model MEMP(20) at 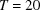
 K; (*b*) model MIAM(20) at 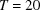
 K; (*c*) model MINV(20) at 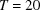
 K. The structure model with ellipsoid representation of the atomic displacement parameters of the atoms is superimposed in each case.

MEM electron densities are dynamic electron densities. Ideally, the MEM produces an unbiased electron density map corresponding to the data. In practice, the MEM electron density will depend to some extent on the choice of prior density (Prathapa *et al.*, 2013 ▸). For computation of the ESP the MEM electron density needs to be combined with a dynamic nuclear density, for which the structure model underlying the prior density provides the natural choice. Here, we have computed MEM electron densities at 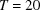
 K for different prior densities. The resulting dynamic ESPs possess similar properties on the 0.5 e Å^−3^ isosurfaces of the electron densities (Table 2 ▸ and Figs. 5 ▸ and 6 ▸ *a*) as well as similar global minimum and maximum values (Table 3 ▸). These similar features indicate that the MEM electron densities conform to the diffraction data with only a weak dependence on the prior. A topological analysis of MEM electron densities has shown a clear but weak dependence on the choice of prior of the MEM electron density at BCPs of covalent bonds (Prathapa *et al.*, 2013 ▸). The latter correspond to density values typically between 2 and 3 e Å^−3^, which is substantially higher that the present isosurface of 0.5 e Å^−3^. Major differences have been found for the Laplacian at BCPs, with positive values in the case of IAM priors and negative values in the case of multipole and invariom priors.

Comparing values of the ESP based on MEM electron densities with those for models shows that the maximum close to the nuclei is nearly identical for the two types of maps. This can be understood from the fact that strongly positive values of ESPs are dominated by contributions from the positive nuclei, and this contribution is identical for the MEM ESP and the model ESP, where the model is the one employed for computation of the nuclear contributions to the MEM ESP. Minimum values of the ESP are found in regions far away from the molecules. They are about twice as negative for the MEM ESP as for the ESP from model densities (Table 3 ▸). This is in agreement with the additional smearing of MEM densities as compared to structure models, always resulting in a higher electron density of the MEM density within the regions of lowest density. Significant differences between MEM ESP and model ESP are also found for the ESP on the 0.5 e Å^−3^ isosurface (Fig. 5 ▸). The range 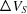 is about 67% larger for MEMAH(20) than for AH(20), with correspondingly larger values for variances and average ESP (Table 2 ▸). These differences also reflect the additional smearing of the electron density in MEM maps. Apart from this scaling, the general features of the MEM ESP are the same as for the model ESPs and the static ESPs, with the most negative values near O atoms and the most positive values near H atoms.

**Figure 6:**
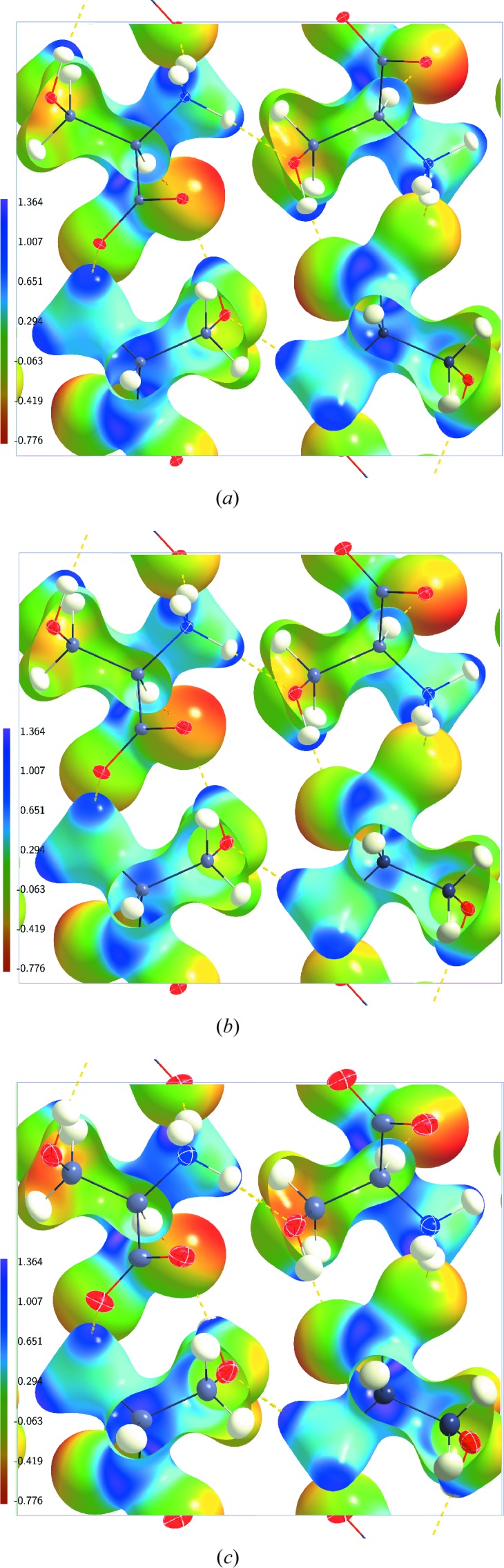
Electrostatic potential (e Å^−1^) of dynamic charge densities of dl-serine mapped on dynamic electron density isosurfaces at 0.5 e Å^−3^. (*a*) Model MEMAH(20) at 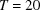
 K; (*b*) model MEMAH(100) at 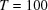
 K; (*c*) model MEMAH(298) at 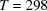
 K. The structure model with ellipsoid representation of the atomic displacement parameters of the atoms is superimposed in each case.

The temperature dependence of the MEM ESP is most pronounced near the nuclei, where with increasing temperature the reduction of the ESP exactly follows the behaviour observed for the corresponding models, as explained above (Table 3 ▸). Any temperature dependence of the other values is much smaller than the difference between the MEM ESP and the model ESP. In particular, the ESPs on 0.5 e Å^−3^ isosurfaces are nearly identical for MEMAH(20), MEMAH(100) and MEMAH(298) (Fig. 6 ▸ and Table 2 ▸).

## 5. Conclusions   

We have defined the electrostatic potential (ESP) for dynamic charge densities. A method is proposed for the computation of this dynamic ESP for multipole models and electron densities derived by the MEM. In particular, it is shown that the reciprocal-space summation defining the ESP converges sufficiently fast for dynamic charge densities, because of the presence of a factor with a Gaussian dependence on the length of the scattering vector, as it is provided by the Debye–Waller factor. This method is implemented in a new module, *dESP*, of the *BayMEM* software package (van Smaalen *et al.*, 2003 ▸).

The dynamic ESP has been obtained for various models and MEM densities of dl-serine at temperatures of 20, 100 and 298 K, employing three sets of high-resolution X-ray diffraction data taken from the literature (Dittrich *et al.*, 2005 ▸). It is found that at all temperatures the ESPs of all static and dynamic charge densities possess similar features on the 0.5 e Å^−3^ isosurfaces of the electron densities: the most negative values appear near O atoms and the most positive values appear near H atoms. These features are in agreement with the presence of intermolecular hydrogen bonds in the crystal, and they are in line with similar features observed for static ESPs of other molecules (Kalaiarasi *et al.*, 2016 ▸; Niranjana Devi *et al.*, 2017 ▸; Zhurova *et al.*, 2016 ▸). Major differences between ESPs on these isosurfaces are found between MEM densities and model densities, with a 60% larger range of values 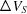 for the MEM densities.

The ESP exhibits only a weak temperature dependence on the 0.5 e Å^−3^ isosurfaces of the dynamic charge densities (Table 2 ▸). A significant reduction of the ESP with increasing temperature is found near the nuclei, where this reduction reflects the increased smearing of the positive charge density of the relevant nucleus (Table 3 ▸). Large differences are found between ESPs of dynamic and static charge densities, since the zero-point thermal smearing is already sufficient to remove the spike at the nucleus in the static electron density.

## Figures and Tables

**Figure 4:**
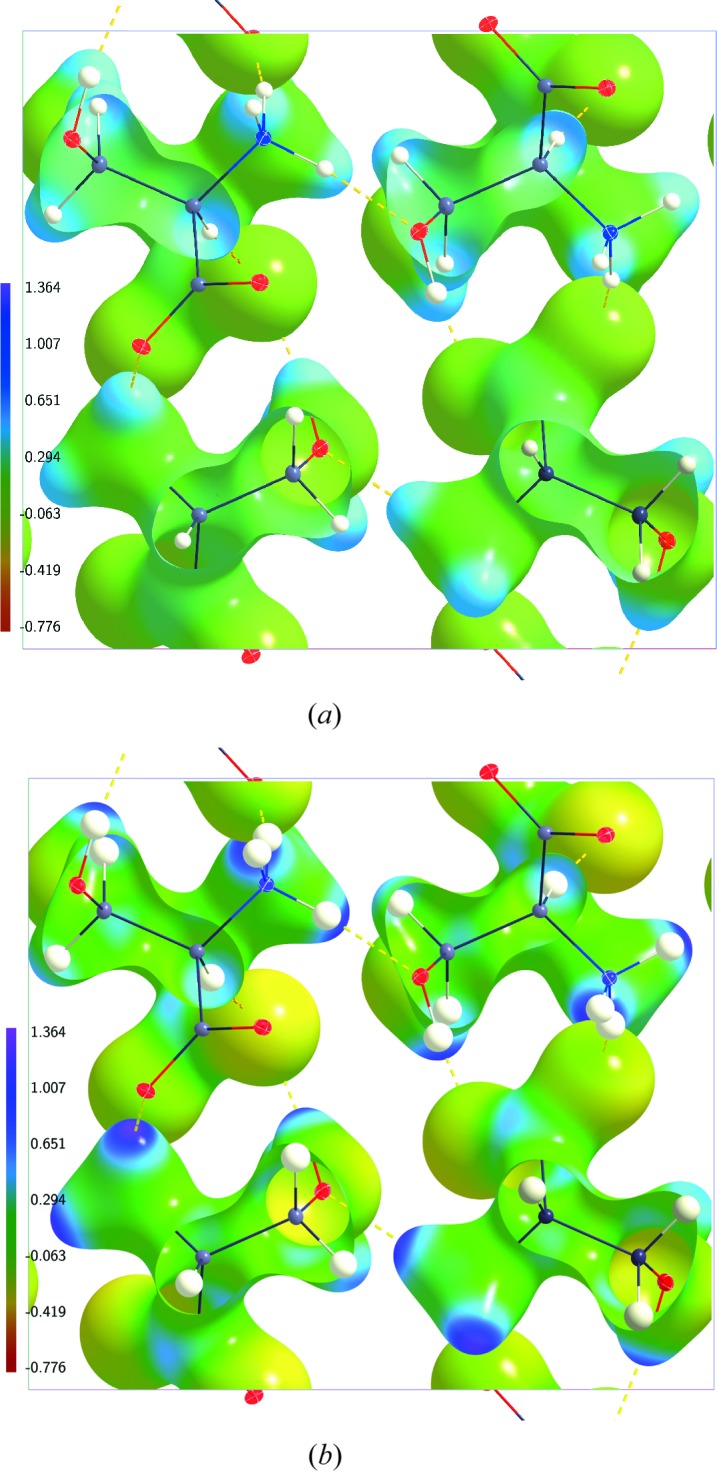
Electrostatic potential (e Å^−1^) of dynamic charge densities of dl-serine mapped on dynamic electron density isosurfaces at 0.5 e Å^−3^. (*a*) Model IAM(20) at 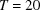
 K; (*b*) model INV(20) at 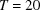
 K. The structure model with ellipsoid representation of the atomic displacement parameters of the atoms is superimposed in each case.

**Table 1:** Definitions of integral quantities of the ESP, integrated over isosurfaces of the electron density according to Politzer *et al.* (2001 ▸)

Symbol	Description
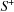	Percentage of surface area with positive ESP
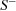	Percentage of surface area with negative ESP
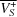	Average ESP, averaged over positive regions of the ESP
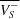	Average ESP, averaged over negative regions of the ESP
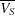	Average ESP, averaged over the entire surface
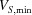	Minimum value of the ESP on the surface
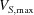	Maximum value of the ESP on the surface
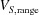	Difference between maximum and minimum values of the ESP on the surface
Π	Average deviation of the ESP from its average value 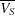
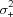	Variance of the ESP over its positive regions
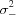	Variance of the ESP over its negative regions
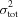	Sum of positive and negative variances of the ESP
ν	Degree of the electrostatic balance derived from the variances of the ESP

**Table 2:** Computed surface quantities of the electrostatic potential of DL-serine mapped on the electron density isosurface at 0.5 e Å^−3^ potential values

Density	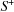 (%)	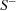 (%)	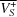 (e Å^−1^)	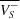 (e Å^−1^)	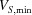 (e Å^−1^)	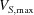 (e Å^−1^)	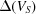 (e Å^−1^)	Π (e Å^−1^)	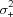 (e^2^ Å^−2^)	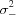 (e^2^ Å^−2^)	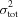 (e^2^ Å^−2^)	ν
Molecule	82.2	17.8	0.548	−0.072	−0.156	1.507	1.663	0.313	0.1015	0.0015	0.1030	0.014331
27 unit cells	100.0	0.0	0.502	0.000	0.025	1.319	1.294	0.227	0.0773	0.0000	0.0773	0.000000
AH(20)	59.8	40.2	0.271	−0.190	−0.384	0.831	1.215	0.228	0.0400	0.0094	0.0493	0.153922
AH(100)	59.9	40.1	0.267	−0.192	−0.381	0.844	1.225	0.227	0.0397	0.0091	0.0488	0.151993 ▸
AH(298)	59.9	40.1	0.260	−0.183	−0.364	0.844	1.208	0.219	0.0367	0.0080	0.0447	0.146735
MP(20)	59.9	40.1	0.271	−0.193	−0.386	0.901	1.287	0.229	0.0403	0.0092	0.0495	0.151864
MP(100)	59.9	40.1	0.266	−0.194	−0.383	0.894	1.277	0.227	0.0401	0.0090	0.0491	0.149382
MP(298)	59.7	40.3	0.259	−0.183	−0.366	0.871	1.237	0.219	0.0363	0.0079	0.0441	0.146285
IAM(20)	65.7	34.3	0.206	−0.079	−0.130	0.582	0.712	0.149	0.0227	0.0008	0.0236	0.033956
INV(20)	56.8	43.2	0.214	−0.162	−0.323	1.102	1.425	0.191	0.0489	0.0070	0.0559	0.109756
MEMAH(20)	62.8	37.2	0.452	−0.229	−0.776	1.260	2.036	0.347	0.0791	0.0268	0.1059	0.189117
MEMAH(100)	59.6	40.4	0.385	−0.233	−0.727	1.153	1.880	0.314	0.0649	0.0268	0.0917	0.206796
MEMAH(298)	60.3	39.7	0.487	−0.255	−0.773	1.364	2.137	0.382	0.0977	0.0324	0.1300	0.187009
MEMP(20)	63.3	36.7	0.451	−0.232	−0.778	1.268	2.046	0.347	0.0815	0.0273	0.1088	0.187906
MEMP(20)sn	62.9	37.1	0.455	−0.228	−0.776	1.266	2.042	0.348	0.0789	0.0269	0.1058	0.189794
MIAM(20)	69.0	31.0	0.482	−0.209	−0.713	1.342	2.055	0.356	0.0990	0.0249	0.1239	0.160648
MIAM(20)sn	68.0	32.0	0.494	−0.209	−0.731	1.363	2.094	0.359	0.0913	0.0245	0.1157	0.166699
MINV(20)	65.3	34.7	0.462	−0.226	−0.764	1.287	2.051	0.349	0.0834	0.0272	0.1106	0.185595

‘sn’ indicates that, for computation of the ESP, the MEM electron density has been combined with the nuclear model of AH(20) instead of the nuclear model of the respective priors.

**Table 3:** Minimum value (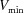) and maximum value (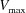) of the ESP (e Å^−1^) Minimum and maximum values have been determined for the ESP given on a grid of mesh 0.035 Å for the molecular and of mesh 0.05 Å for the cluster ESP.

Density	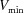	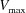
Molecule	−0.26100	520.37
27 unit cells	−0.08775	534.55
AH(20)	−0.51618	46.40
AH(100)	−0.52298	35.07
AH(298)	−0.52243	19.11
MP(20)	−0.51950	46.33
MP(100)	−0.52528	35.11
MP(298)	−0.52470	19.15
IAM(20)	−0.49189	45.17
INV(20)	−0.46899	45.57
MEMAH(20)	−0.91762	46.44
MEMAH(100)	−0.83758	35.31
MEMAH(298)	−0.91645	19.50
MEMP(20)	−0.92624	46.40
MEMP(20)sn	−0.92119	46.46
MIAM(20)	−0.95602	44.95
MIAM(20)sn	−0.95521	47.06
MINV(20)	−0.94533	45.62

## Appendix A Surface quantities

The ESP is analysed through its values on an isosurface of the electron density. Quantitative measures are obtained as integral properties, integrated over this surface (Politzer *et al.*, 2001 ▸). Definitions of the properties are given in Table 1 ▸. Here we give the mathematical formulae, used to compute these quantities. The electron density and the ESP are given by their values on a grid over the unit cell. α and β indicate the number of surface pixels with positive and negative values of the ESP, respectively, while . Then,
